# *Technomyrmex montaseri* sp. n., a new ant species  of the *T. gibbosus*-group from Oman (Hymenoptera, Formicidae) with a key to the *Technomyrmex* species of the Arabian Peninsula

**DOI:** 10.3897/zookeys.108.930

**Published:** 2011-06-17

**Authors:** Mostafa R. Sharaf, Cedric A. Collingwood, Abdulrahman S. Aldawood

**Affiliations:** 1 Plant Protection Department, College of Food Sciences and Agriculture, King Saud University, Riyadh 11451, P. O. Box 2460. Saudi Arabia; 2 18 Milton Street, Skipton, North Yorkshire, BD23 2ED, UK

**Keywords:** *Technomyrmex*, Palaearctic, Middle East, Alpha taxonomy, Arabia, Key

## Abstract

*Technomyrmex montaseri* **sp. n.** is described and illustrated from Oman based on the worker caste collected in Bani Sur. It belongs to the *Technomyrmex gibbosus*-group, with closest resemblance to *Technomyrmex vexatus* (Santschi, 1919) and *Technomyrmex gibbosus* W. M. Wheeler, 1906. A key to the Arabian *Technomyrmex* is given.

## Introduction

The ant genus *Technomyrmex* Mayr, 1872 comprises one of the largest and most diverse ant genera in the subfamily Dolichoderinae. Ninety species are known world wide, distributed throughout the tropical and subtropical zones; most species occur in the Oriental-Malesian (*sensu* Bolton 2007) and Afrotropical regions. The workers of the genus *Technomyrmex* are clearly diagnosed by the following characters (as defined by Bolton 2007). Masticatory margin of mandible multidentate, with 12-14 teeth. Palp formula 6,4 in the vast majority of species. Median portion of anterior clypeal margin transverse to very deeply incised. Antennae 12-segmented, without a club. Metanotal groove present. Propodeum unarmed, its dorsum-declivity junction broadly rounded to distinctly angular. Petiole extremely reduced, forming a low narrow segment without a node or scale. Petiole concealed in dorsal view when gaster is in line with mesosoma, overhung by the anteriorly projecting first gastral tergite. A groove is present in the anterior face of the first gastral tergite that accommodates the petiole. Gaster with five visible tergites and sternites, the pygidium small.

The *Technomyrmex* of the Arabian peninsula have been studied fragmentarily and probably many species remain undiscovered. This paper continues our contributions towards the knowledge of Arabian *Technomyrmex*. The first contribution was by Collingwood (1985). He reported *Technomyrmex albipes* (Smith, 1862) and *Technomyrmex* sp. A, which is almost certainly a member of the genus *Tapinoma* (fig. 14), *Technomyrmex* sp. B and described *Technomyrmex setosus*. The *Technomyrmex* sp. B was compared with *Technomyrmex gibbosus* W. M. Wheeler, 1906 and it was felt both sp. A and sp. B might well prove to be new species. The illustrations of *Technomyrmex* species A and B are mislabelled in Figs. 12-14, with 14 being the smaller-eyed species A and 13 being species B. Collingwood and Agosti (1996) followed this by recording the same species and adding *Technomyrmex bruneipes* Forel, 1895 from Yemen and other localities in Saudi Arabia. An additional record of *Technomyrmex* sp. B was from Oman and Yemen. Bolton (2007) reverted *Technomyrmex bruneipes* to the status of a junior synonym of *Technomyrmex albipes*. More recently, Sharaf (2009) described a further member of the *Technomyrmex albipes*-group, *Technomyrmex briani* from Wadi Abha, Asir, Saudi Arabia. With the revision of *Technomyrmex* by Bolton (2007), we have been able to confirm *Technomyrmex* sp. B as a new species.

The *Technomyrmex gibbosus* group (Bolton 2007) is distinguished from other *Technomyrmex* species-groups by the combination of the following characters. The anterior clypeal margin has only the weakest of median impressions and setae are entirely lacking from the head behind the clypeus, the mesosoma including the propodeal declivity, and gastral tergites 1-3. With the mesosoma in profile the pronotum and mesonotum form separate curved surfaces and the mesonotum is distinctly convex. Palp formula 6,4. Bolton gave only two species in the group, *Technomyrmex gibbosus* from Japan and *Technomyrmex vexatus* from Morocco and recently recorded from the Iberian Peninsula (Gibraltar) (Guillem and Bensusan 2008). The latter and *Technomyrmex setosus* are the only species he listed from the “Western Palaearctic”, a geographical area in which he includes Saudi Arabia and Yemen. Bolton was unable to locate the Collingwood type specimens and noted those he examined from the Liverpool Museum matched the Collingwood description but not the drawing.

In this paper *Technomyrmex montaseri* new species is described from Oman.

## Material and methods

Measurements and indices were taken according toBolton (2007).

### Measurements:

TL*Total Length.* The total outstretched length of the ant from the mandibular apex to the gastral apex.

HL*Head Length*. The length of the head capsule excluding the mandibles; measured in full-face view in a straight line from the mid-point of the anterior clypeal margin to the mid-point of the posterior margin. In species where one or both of these margins is concave the measurement is taken from the mid-point of a transverse line that spans the apices of the projecting portions.

HW*Head width*. The maximum width of the head behind the eyes, measured in full-face view.

SL*Scape length*. The maximum straight-line length of the scape, excluding the basal constriction or neck that occurs just distal of the condylar bulb.

PW*Pronotal width*. The maximum width of the pronotum in dorsal view.

WL*Weberś length of Mesosoma*. The diagonal length of the mesosoma in profile, from the anterior most point of the pronotum to the posterior basal angle of the metapleuron.

All measurements are expressed in millimeters.

### Indices:

CI*Cephalic Index*. HW divided by HL, × 100.

SI*Scape Index*. SL divided by HW, × 100.

OI*Ocular Index*. Maximum diameter of eye divided by HW, × 100.

EPI*Eye Position Index*. In full-face view the straight-line length (parallel to the long axis of the head) from the anteriormost point of the eye to the anterior clypeal margin, divided by the straight-line length from the posteriormost point of the eye to the posterior margin, × 100.

DTI*Dorsal Thoracic Index*. In dorsal view the length from the mid-point of the anterior pronotal margin to the midpoint of the metanotal groove, divided by PW, × 100.

The photographic images were taken using a digital camera attached to a stereomicroscope. The microscope was equipped with a Z-Stepper to enable the generation of usually 30 images in different focus layers from which a montage image was computed using Auto-Montage Pro.

## Results

### Technomyrmex montaseri sp. n.

urn:lsid:zoobank.org:act:AC02AAFB-ACF2-4491-9E11-401DE099AC84

http://species-id.net/wiki/Technomyrmex_montaseri

Figs 1- [Fig F2] 3 

#### Holotype worker.

Oman, Bani Sur, 7.iii.1984 (W. Büttiker); the entomological Collection, the World Museum Liverpool (WML), Liverpool, U.K. deposited by Mr Guy T. Knight)

#### Paratypes.

7 workers with same data as holotype in the World Museum, Liverpool (WML) (deposited by the senior author), 1 worker with same data as holotype, The Natural History Museum, London (BMNH) (deposited by B. Bolton) and 1 worker, Oman Eastern sand project (Leg. Collingwood) at WML.

#### Holotype worker.

*Measurements*: TL: 2.90; HL: 0.60; HW: 0.57; SL: 0.62; PW: 0.37; WL: 0.80; EL: 0.15; *Indices*: CI: 95; SI: 109; OI: 26; EPI: 80; DTI: 122

#### Paratype worker.

*Measurements*: TL: 2.80; HL: 0.62; HW: 0.60; SL: 0.58; PW: 0.38; WL:0.65; EL: 0.15; *Indices*: CI: 97; SI: 97; OI: 25; EPI: 125; DTI: 126

Other workers, as *Technomyrmex* species B, measurements given by Collingwood (1985) are as follows: TL:2.20, HL:0.52; HW:0.52; SL:0.62; EL: 0.16; SI:119

#### Distribution.

Saudi Arabia, Al Farrash, 15.x.1982, 21° 7’ 45 N, 40° 36’ 20 E; collector W. Büttiker (Collingwood 1985); Oman, Bani Sur (as the holotype and paratype) and Eastern Sands, iii.1986, all W. Büttiker; also, Yemen, Al-Hajjarah, 14.iii.1992, A van Harten (Collingwood and Agosti 1996).

#### Diagnosis.

This new species is characterized by the combination of the following characters: Head, mesosoma and all gastral tergites without setae. Anterior clypeal margin with a shallow but distinct median concavity. In full-face view the occipital margin and the sides of the head are convex.

#### Worker description.

Dorsum of head behind clypeus entirely lacks setae. Anterior clypeal margin with a shallow but distinct median concavity. In full-face view the posterior margin of the head and the sides clearly convex. Eyes of moderate size, located in front of the midlength and their outer margins just failing to break the outline of the sides. Sculpture of head a very weak, superficial and effaced microreticulum. Dorsum of mesosoma and propodeal declivity entirely lack setae. With mesosoma in profile the mesonotal dorsal outline consists of an anterior section that is short and flat to feebly convex; posterior to this the surface curves broadly and evenly into a larger, more steeply sloped posterior section that descends to the narrow mesonotal groove. Propodeum in profile with a short convex dorsal surface that rounds evenly into the declivity, the two surfaces not separating by an angle. Sculpture reduced and superficial on dorsal mesosoma and all gastral tergites; the latter without pubescence. All gastral tergites, scapes and tibiae without setae. Colour uniform yellow.

#### Derivatio nominis.

A patronymic name (*Technomyrmex montaseri*) is proposed in honor of Mosrafa Sharaf's friend the famous Egyptian journalist Mr. Salah Montaser (Al-Ahram News paper).

**Figure 1.**
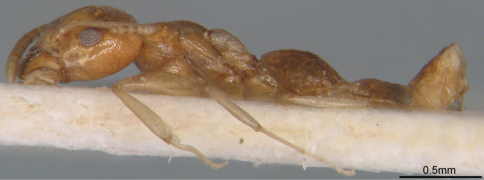
Profile of *Technomyrmex montaseri* sp. n. (Holotype, WML).

**Figure 2. F2:**
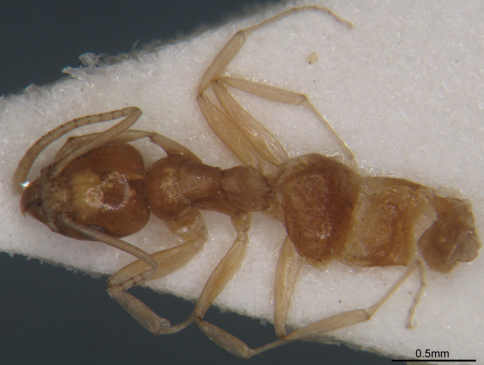
Dorsal view of *Technomyrmex montaseri* sp. n. (Holotype, WML).

**Figure 3.**
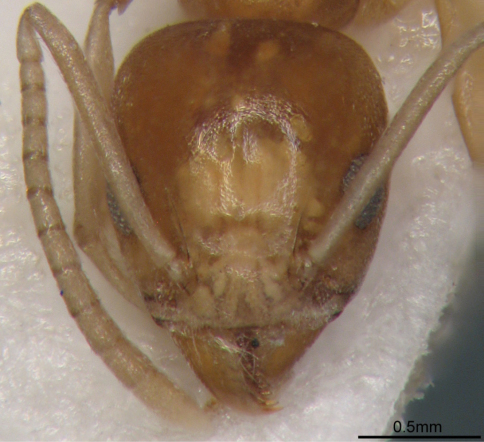
Full-face view of *Technomyrmex montaseri* sp. n. (Holotype, WML)

## Key to the Arabian species of the genus *Technomyrmex*

We include *Technomyrmex vexatus* in the key of the Arabian *Technomyrmex* as it is the only other West Palaearctic species of its group and may well be discovered in other areas, beside Morocco and Gibraltar where it now is known.

**Table:** 

1	With the head in profile the dorsal surface of the frontal carina, or the dorsum immediately mesad of the frontal carina, entirely without setae	2
–	With the head in profile the dorsal surface of the frontal carina, or the dorsum immediately mesad of the frontal carina, with setae present; at least with one seta present somewhere along the length of the frontal carina, or more usually with a row of 2-4	3
2	Head and mesosoma brown. Posterior margin of head shallowly impressed, eyes located close to the midlength of the head; the gastral tergites 1-3 without setae, the fourth with 2-3 pairs. Larger species with TL 3.0-3.4, EPI 68-76, WL 0.90-0.96 (Spain, Morocco and possibly in Arabia)	*Technomyrmex vexatus*
–	Head and mesosoma yellow. Posterior margin of head clearly convex, eyes located in front of the midlength of the head; all gastral tergites without setae. Smaller species with TL 2.2-2.9, EPI 80-125, WL 0.65-0.80 (Oman, Yemen and Saudi Arabia)	T. montaseri sp. n.
3	Gastral tergites 2-3 without setae (Saudi Arabia)	*Technomyrmex brianis*
–	Gastral tergites 2-3 with setae present; setae may be restricted to one or two pairs on each segment or may be numerous	4
4	With head in profile the dorsum behind the level of the posterior margin of the eye without setae. Head, mesosoma, petiole and gaster blackish brown to black. Propodeum dorsum-declivity junction distinctly and sharply angled. HL 0.56-0.63, WL 0.66-0.78, SI 91-102 (Successful tramp species)	*Technomyrmex albipes*
–	With the head in profile the dorsum behind the level of the posterior margin of the eye with one or more pairs of setae present, which may be very short and inconspicuous. Head and gaster brown to dark brown; mesosoma a much lighter yellowish brown and distinctly contrasting. Propodeum dorsum-declivity junction broadly rounded or very weakly angled. HL 0.64-0.67, WL 0.80-0.82, SI 108-112 (Saudi Arabia)	*Technomyrmex setosus*

## Discussion

### Affinities

This new species is a member of the *Technomyrmex gibbosus*-group as defined by Bolton (2007) and cannot be identified with any of the *Technomyrmex* species in his extensive review. *Technomyrmex montaseri* appears taxonomically closest to *Technomyrmex vexatus* (Santschi, 1919), known only from Morocco and Gibraltar, and *Technomyrmex gibbosus* W. M. Wheeler, 1906, which was described from Japan and otherwise know only from North Korea (Radchenko 2005). All three species are completely without setae on the head behind the clypeus, or on the mesosoma including the propodeal declivity. With the mesosoma in profile the mesonotal dorsal outline is convex, consisting of a shallowly convex anterior section that curves broadly and evenly into a more sloping shallow convexity that descends to the metanotal groove. The propodeum in profile has a short convex dorsal surface that rounds into the declivity. Scapes and tibiae without setae. *Technomyrmex montaseri* may be closer to *Technomyrmex vexatus*, but differs in colour which is uniform yellow while it is brown in *Technomyrmex vexatus*. *Technomyrmex montaseri* also is consistently smaller, TL 2.8-2.9 versus TL 3.-3.4. *Technomyrmex montaseri* has the eyes located in front of the midlength of the head, whereas in *Technomyrmex vexatus* they are situated close to the midlength; thus the eye position index is larger, EPI 80-125, versus EPI 68-76. *Technomyrmex montaseri* has a higher scape index, SI 97-109 versus SI 90-94; has a smaller Weber’s length of mesosoma, WL 0.65-0.80 versus WL 0.90-0.96, and has a clearly convex occipital margin, that is very shallowly impressed in *Technomyrmex vexatus*. Additionally, *Technomyrmex montaseri* has completely bare gastral tergites, while in *Technomyrmex vexatus* gastral tergites 1-3 are without setae but the fourth has 2-3 pairs. *Technomyrmex montaseri* lacks pubescence on the first gastral tergite, whereas short and sparse pubescence is present in *Technomyrmex vexatus*.

*Technomyrmex montaseri* and *Technomyrmex gibbosus* are similar in most measurements but the scape length in *Technomyrmex montaseri* is consistently larger, SL 0.58-0.62 versus SL 0.50-0.54. *Technomyrmex montaseri* has a higher cephalic index, CI 95-97 versus SI 86-91, a higher scape index, SI 97-109 versus SI 85-93; a significantly higher eye position index, EPI 80-125 versus EPI 50-58; and a smaller Weber’s length of mesosoma, WL 0.65-0.80 versus WL 0.76-0.84. In *Technomyrmex montaseri* the posterior margin of the head and the sides are broadly convex, whereas in *Technomyrmex gibbosus* the posterior margin of the head has a median indentation and the sides are only shallowly convex. In *Technomyrmex montaseri* all gastral tergites are bare whereas in *Technomyrmex gibbosus* gastral tergites 1-3 lack setae but the fourth tergite has 1-2 pairs. *Technomyrmex montaseri* is yellow while *Technomyrmex gibbosus* has a medium to dark brown mesosoma, often with a reddish tint; the gasters are about the same medium to dark brown, with the legs dull yellow to yellowish brown. Moreover, *Technomyrmex gibbosus* has very fine, short, appressed pubescence present on the first and second gastral tergites. This pubescence is somewhat more dense in *Technomyrmex gibbosus* than *Technomyrmex vexatus*.

### Technomyrmex gibbosus-group

As previously known, the *Technomyrmex gibbosus*-group contains a pair of geographically widely separated species, *Technomyrmex vexatus* from Morocco and Gibraltar and *Technomyrmex gibbosus* from North Korea and Japan. The distribution of *Technomyrmex montaseri* (Oman, Yemen and Saudi Arabia) fills a gap in that distribution. Bolton (2007: 41, 82) speculated that *vexatus* and *gibbosus* might share an intermediate common ancestor, or could be remnants of a fairly distinctive species group that once extended across the width of the southern Palaearctic. Alternatively each might have acquired the shared characters by convergence from unrelated ancestors. With our specimens of this new species we feel able to support the idea of the intermediate common ancestor from Western or Central Asia.

Five species of the genus *Technomyrmex* are known now from the Arabian Peninsula. If one takes into account the large area and its location between the Palaearctic, Oriental and Afrotropical regions, this figure is very low. Therefore, one would expect to find many more species of this genus and from the *gibbosus*-group with more intensive collecting efforts.

## Supplementary Material

XML Treatment for Technomyrmex montaseri
